# Aplastic Anemia Treated with Eltrombopag during Pregnancy

**DOI:** 10.1155/2022/5889427

**Published:** 2022-02-25

**Authors:** Yuri Suminaga, Yoshitsugu Chigusa, Tadakazu Kondo, Haruko Okamoto, Yosuke Kawamura, Mana Taki, Masaki Mandai, Haruta Mogami

**Affiliations:** ^1^Department of Gynecology and Obstetrics, Kyoto University, 54 Shogoin Kawahara-cho, Sakyo-ku, Kyoto 606-8507, Japan; ^2^Department of Hematology and Oncology, Kyoto University, 54 Shogoin Kawahara-cho, Sakyo-ku, Kyoto 606-8507, Japan

## Abstract

Aplastic anemia is a rare blood disorder characterized by pancytopenia and hypocellular bone marrow. In patients with aplastic anemia, pancytopenia sometimes worsens during pregnancy, and relapse of aplastic anemia in pregnancy is common. Nevertheless, only supportive care with blood products is the mainstay of treatment of aplastic anemia in pregnancy. Thus, the obstetric management and treatment of aplastic anemia in pregnancy is extremely challenging. We herein report the first case of a pregnant woman complicated with aplastic anemia who was successfully treated with eltrombopag, a thrombopoietin receptor agonist. A 27-year-old primigravida woman who had a history of aplastic anemia refractory to immunosuppressive therapy and was treated with eltrombopag became pregnant. Eltrombopag treatment was continued after weighing the benefits and potential risks. Throughout pregnancy, the woman's pancytopenia did not progress, and she delivered a 2336 g baby vaginally at 38 weeks of gestation. Her postpartum outcome was uneventful, and the neonate did not develop thrombocytosis. Since the efficacy and safety of eltrombopag in pregnancy has not yet been established, its routine use should be avoided. However, if limited to refractory cases and with adequate maternal and fetal monitoring, including neonatal blood examinations, the use of eltrombopag for patients with aplastic anemia during pregnancy may be acceptable and result in favorable maternal and fetal outcomes.

## 1. Introduction

Aplastic anemia is a rare and heterogeneous blood disorder characterized by pancytopenia and hypocellular bone marrow [1]. Aplastic anemia causes anemia, bleeding, and infection due to associated cytopenia, and these complications can sometimes be life-threatening, especially during pregnancy. Generally, pancytopenia can often progress during pregnancy [1], and relapse of aplastic anemia is common in pregnancy in patients previously treated with immunosuppressive therapy [2]. Therefore, together with the paucity of cases and limited treatment choices, it remains challenging to manage a pregnancy complicated by aplastic anemia.

Eltrombopag, a thrombopoietin receptor agonist, acts as a thrombopoietin mimetic and leads to increased platelet counts in patients with immune thrombocytopenia (ITP). In a phase 2 study involving patients with refracted aplastic anemia, eltrombopag produced a hematologic response in at least one lineage in 11 of 25 patients (44%) [3]. Moreover, in its follow-up study, eltrombopag eventually increased in neutrophil, red blood cell, and platelet lineages in 7 of 43 patients (16%) [4]. Thus, eltrombopag is a promising option for the treatment of aplastic anemia. However, thus far, there have been no reports in which patients with aplastic anemia were treated with eltrombopag during pregnancy, although it has been used in limited cases for patients with ITP in pregnancy [5, 6].

We herein show the case of a pregnant woman with aplastic anemia who continued eltrombopag treatment throughout pregnancy, and maternal and neonatal outcomes were favorable.

## 2. Case Report

The patient was a 27-year-old primigravida woman. She was diagnosed with aplastic anemia by bone marrow examination at the age of 20. She had been in remission with the treatment of antithymocyte globulin (ATG) and cyclosporine. At the age of 24, however, pancytopenia progressed (Table 1), and eltrombopag was introduced at 25 mg/day and soon increased to 50 mg/day, in addition to cyclosporine at 50 mg/day. She achieved a trilineage response, and she continued to show hematologic improvement. At the age of 27, she became pregnant, and cyclosporine treatment was ceased at her request. Her hematologist explained that the usage of eltrombopag during pregnancy was limited, but the treatment of ITP with eltrombopag during pregnancy showed no maternal complications or fetal abnormalities, and the benefits of using eltrombopag would overcome the disadvantages. She decided to continue taking eltrombopag, and the dose was reduced to 25 mg/day because her condition was stable. In the first trimester of pregnancy, she could not take eltrombopag for three weeks due to hyperemesis gravidarum, and her platelet count slightly decreased (Table 1). After the improvement of hyperemesis gravidarum, she was able to resume taking eltrombopag, her platelet count recovered, and her pregnancy course was stable thereafter. At 37^+2^ weeks of gestation, her systolic blood pressure rose to 130 mmHg, and a urinalysis showed proteinuria (+). In addition, a fetal ultrasound showed mild fetal growth restriction: the baby's estimated body weight was less than the 10^th^ percentile. Thus, the patient was admitted to the hospital for enhanced monitoring of the mother and baby. Although there was no increase in her blood pressure after admission, her platelet count at 38^+5^ weeks of gestation was 57 × 10^9^/L with a downward trend. Due to the indications of mild fetal growth restriction, comparably high blood pressure, and maternal thrombocytopenia, we decided to induce labor. She vaginally delivered a mature female infant weighing 2336 g (5.4^th^ percentile) with an Apgar score of 9 at 1 minute and 5 minutes. The blood loss at delivery was 680 grams, the delivery was uneventful, and no blood transfusion was required. The infant was formula-fed. The results of the newborn's blood tests were all normal (Table 2). Pathological examination of the placenta revealed one small infarction in the villi. Microscopically, perivillous fibrin deposition was prominent. In addition, fibrous thickening and obliteration at stem vessels were observed, which indicated fetal vascular malperfusion. These pathological findings are consistent with fetal growth restriction. The mother's postpartum course was uneventful, and her remission was maintained by 25 mg of eltrombopag alone.

## 3. Discussion

The first case of aplastic anemia described in the literature was a pregnant woman who died of postpartum hemorrhage in 1888. Although there has been no proven causal association between pregnancy and aplastic anemia, it cannot be ruled out that pregnancy may have some influence on the disease [7]. In some cases, women with aplastic anemia experience worsening symptoms or relapse during pregnancy [1, 2], and marked thrombocytopenia is often observed with advancing gestation. However, available treatment options during pregnancy are limited; hematopoietic stem-cell transplantation or the use of ATG is generally contraindicated in pregnancy [1, 8], and only cyclosporin, whether it is effective or not, is thought to be safe. Ultimately, supportive care with blood products is the mainstay of treatment of aplastic anemia in pregnancy. Thus, an effective treatment that can be used during pregnancy is needed.

Eltrombopag is a relatively new drug for the treatment of steroid-refractory ITP and severe aplastic anemia that is refractory to immunosuppressive therapy. Eltrombopag binds to the thrombopoietin receptor on megakaryocytes and hematopoietic stem cells, which results in platelet production and overcoming the depletion of hematopoietic stem cells in aplastic anemia [3]. The Food and Drug Administration assigned eltrombopag to pregnancy risk category C. This means that eltrombopag, although its use is not contraindicated, should be administered very carefully during pregnancy, only when its benefits outweigh its potential risks, because there are no adequate and well-controlled studies in humans, and eltrombopag is thought to cross the placenta. The patient in this case had been refractory to immunosuppressive therapy and achieved remission by eltrombopag. In other words, the risk of pancytopenia was extremely high if eltrombopag treatment was discontinued. Moreover, the worsening of aplastic anemia during pregnancy has been shown to be associated not only with disease complications but also with pregnancy-related complications [9]. Therefore, it was not advisable for her to discontinue the use of eltrombopag, and after obtaining her informed consent, eltrombopag treatment was continued during pregnancy.

At present, no marked maternal or fetal toxicity of eltrombopag has been reported in animal studies at drug doses equivalent to those given to humans [6]. Case series and case reports have been published in which eltrombopag was administered to pregnant women with ITP [5, 6]. According to these studies, the platelet response to eltrombopag was favorable in most cases, and significant maternal and neonatal complications that could be clearly attributed to eltrombopag were not reported. There have also been published case reports of the successful treatment of severe thrombocytopenia with eltrombopag during pregnancy in patient with systemic lupus erythematosus [10] or MYH9-related disease, an autosomal dominant thrombocytopenia [11]. In these literatures, no short-term adverse effects of eltrombopag on newborns have been reported. Nonetheless, eltrombopag could theoretically cross the placenta [12, 13], and potentially affect the fetus. In the present case, we assessed a complete blood count of the neonate for 3 months after birth and confirmed that there was no abnormal increase in trilineage. Michel et al. reported one neonate with thrombocytosis that was born to a mother taking 100 mg of eltrombopag due to ITP for 4 weeks beginning at 30 weeks of gestation [5]. Careful observation and blood examinations are necessary for neonates born to mothers who receive eltrombopag treatment during pregnancy.

Currently, there are no data on the safety of breastfeeding while taking eltrombopag, and the patient, in our case, discontinued breastfeeding. However, given that the molecular weight of eltrombopag is 445.2 [14] and its protein binding rate is more than 99% [15], the secretion of eltrombopag in the milk may be small. Furthermore, calcium in breast milk may attenuate the effect of eltrombopag because the bioavailability of eltrombopag is reduced by the chelation of calcium [16]. In this light, breastfeeding while receiving eltrombopag may not necessarily be contraindicated if the neonate's blood and symptoms such as vomiting, ecchymosis, hepatotoxicity, and conjunctival hemorrhage are adequately monitored, even though it is not recommended in principle.

It is not clear whether the fetal growth restriction seen in our case was directly associated with aplastic anemia or eltrombopag treatment. Other than postpartum hemorrhage, there are no known obstetrical complications that are specific to pregnancy in women with aplastic anemia. Remarkably, however, previous studies have reported that the rate of pregnancy complications in women with aplastic anemia, including preeclampsia, fetal growth restriction, and preterm delivery, were variable [8, 17, 18]. Interestingly, infarction in the placenta without fetal growth restriction has been reported previously in cases of ITP that were treated with the thrombopoietin receptor agonist romiplostim during pregnancy [19]. Given that the thrombopoietin receptor is only slightly expressed in cytotrophoblasts [20], it is unlikely that eltrombopag caused the pathological changes in the placenta, including in our case.

In conclusion, to the best of our knowledge, this is the first case report in which a patient with aplastic anemia was successfully treated with eltrombopag during pregnancy. Since the efficacy and safety of eltrombopag in pregnancy has not yet been established, its routine use should be avoided. However, if limited to refracted cases and with adequate maternal and fetal monitoring, including neonatal blood examinations, the use of eltrombopag for patients with aplastic anemia during pregnancy may be acceptable and result in favorable maternal and fetal outcomes.

## Figures and Tables

**Table 1:** The changes in maternal complete blood counts.

Timing	At the age of 24	Just before conception	11w5d	13w4d	27w2d	37w2d	38w5d	PPD 32
WBC (10^9^/L)	2.88	4.64	4.97	5.84	4.89	4.72	5.32	4.04
Neut (10^9^/L)	1.73	2.74	3.71	4.45	3.75	3.56	3.72	2.30
Hb (g/dL)	7.8	12.9	12.4	13.4	10.1	10.4	10.0	11.6
Ht (%)	23.3	40.3	36.7	39.1	30.9	33.8	33.1	36.4
Reti (10^12^/L)	0.04	0.08	0.07	0.08	0.06	0.10	0.14	0.07
Plt (10^9^/L)	18	98	66	73	65	72	57	125

WBC: white blood cells. Neut: neutrophils. Hb: hemoglobin. Ht: hematocrit. Reti: reticulocytes. Plt: platelets. w: weeks. d: days. PPD: postpartum day.

**Table 2:** The changes in neonatal complete blood counts.

Timing	Day 0^∗^	Day 2	Day 4	Day 95
WBC (10^9^/L)	4.77	6.95	4.67	4.97
Hb (g/dL)	10.9	14.9	14.3	10.2
Ht (%)	32.4	42.0	39.6	30.1
Plt (10^9^/L)	237	197	179	237

WBC: white blood cells. Hb: hemoglobin. Ht: hematocrit. Plt: platelets. ^∗^umbilical vein data.

## Data Availability

The data used to support the findings of this study are included within the article.
